# Colony-Forming Unit Spreadplate Assay versus Liquid Culture Enrichment-Polymerase Chain Reaction Assay for the Detection of *Bacillus* Endospores in Soils

**DOI:** 10.3390/geosciences10010005

**Published:** 2019

**Authors:** Dale W. Griffin, John T. Lisle, David Feldhake, Erin E. Silvestri

**Affiliations:** 1 St. Petersburg Coastal and Marine Science Center, U.S. Geological Survey, 600 4th Street South, St. Petersburg, FL 33772, USA; 2 Pegasus Technical Services, Inc., 46 East Hollister St., Cincinnati, OH 45219, USA; 3 Homeland Security and Materials Management Division, Center for Environmental Solutions and Emergency Response, U.S. Environmental Protection Agency, 26 West Martin Luther King Drive, MS NG16, Cincinnati, OH 45268, USA

**Keywords:** *B. anthracis*, pathogen, soil, detection, polymerase chain reaction, enrichment, inhibition

## Abstract

A liquid culture enrichment-polymerase chain reaction (E-PCR) assay was investigated as a potential tool to overcome inhibition by chemical component, debris, and background biological impurities in soil that were affecting detection assay performance for soil samples containing *Bacillus atrophaeus* subsp. *globigii* (a surrogate for *B. anthracis*). To evaluate this assay, 9 g of matched sets of three different soil types (loamy sand [sand], sandy loam [loam] and clay) was spiked with 0, ~4.5, 45, 225, 675 and 1350 endospores. One matched set was evaluated using a previously published endospore concentration and colony-forming unit spreadplate (CFU-S) assay and the other matched set was evaluated using an E-PCR assay to investigate differences in limits of detection between the two assays. Data illustrated that detection using the CFU-S assay at the 45-endospore spike level started to become sporadic whereas the E-PCR assay produced repeatable detection at the ~4.5-endospore spike concentration. The E-PCR produced an ~2-log increase in sensitivity and required slightly less time to complete than the CFU-S assay. This study also investigated differences in recovery among pure and blended sand and clay soils and found potential activation of *B. anthracis* in predominately clay-based soils.

## 1. Introduction

The known pathogenic species of *Bacillus* are Gram-positive endospore formers that can grow under both aerobic and facultatively anaerobic conditions and are ubiquitous in nature [1]. Pathogenic species include *Bacillus anthracis*, the causative agent of anthrax, *Bacillus cereus*, known to cause diarrhea through food poisoning, and several other species, such as *Bacillus licheniformis*, *B. pumilus*, *B. thuringiensis* and *B. subtilis*, that have also been identified as capable of causing foodborne illness [2]. The primary species of concern, *B. anthracis*, can persist in soil for many years under harsh environmental conditions in endospore form, and causes frequent natural disease outbreaks in agricultural animals and wildlife in several geographic regions in North America, including southern Texas, the tri-state area of Minnesota, South Dakota, North Dakota, and north into Canada. Known influential factors of outbreaks include periods of drying following heavy precipitation events and elevated concentrations of elements in soil such as calcium, phosphate, magnesium, sodium, copper, manganese, strontium and zinc [3–7]. However, more recent research indicates that soil conditions such as soil type, sand content, clay content and regional geochemistry might play a role in the ability of this pathogen to persist once released from a host as an endospore [8–10].

There is a continued need for a sensitive method to detect these pathogens [11,12], especially for soil samples that have been collected in response to a potential naturally occurring outbreak in wildlife, livestock or humans or for bioterror investigations which might contain different types of soil. The ‘ideal’ method for *Bacillus* endospore recovery and identification in soil samples has yet to be identified and has been hindered by recovery and purification issues, limitations of detection assays, and susceptibility of assays to interference from various environmental constituents [8,13–15]. Other issues that can limit the usability of an assay include costs, ease of use, and time to completion. Use of traditional culture methods has been reported for the concentration and growth of endospores in soil. An assay that used spikes of 500 endospores in 7.5 g of soil was able to recover two to three colony-forming units [16]. An assay that utilized endospore spikes in 50 g of two different sterile soil types and utilized matched samples was screened by two laboratories and reported a limit of detection of 14 endospores g^−1^ of soil [15]. However, the use of culture-based assays with nonsterile soil would be hindered by background organisms in the soil which can quickly overwhelm culture plates. In addition, with most traditional culture methods, a limitation is that the identification of colonies is presumptive at best. Molecular assays such as those that use *B. anthracis* specific tagged antibodies or the polymerase chain reaction (PCR) are needed for positive identification. Culture assays can be coupled with molecular assays for rapid positive identification. For example, a rapid viability PCR (RV-PCR) assay was developed using spiked wipes, air filters and water that reported a sensitivity level between 10 and 99 B. *anthracis* endospores [17]. A most probable number (MPN)-PCR method was developed for the detection of *B. anthracis* on surface swabs using spikes of 3 × 10^4^, 400 and 40 endospores [18]. Letant et al. noted that the MPN-PCR data was within 1-log of matched culture data and that the MPN-PCR method ‘*tended to overestimate the expected results, especially at lower spore levels*.’ The benefit of a coupled culture-detection assay is that it enables one to address viability (through enrichment) and potentially, quantitation. However, these culture-detection assays were not developed using soil as a matrix and none of the aforementioned assays investigated how the differences in soil type can affect sample processing and method performance.

The primary objective of this study was to develop an ‘ease of use (minimal handling steps compared to other culture-based PCR methods)’ liquid culture enrichment-presence/absence-PCR (E-PCR) protocol for soil samples in order to try to overcome inhibition by chemical component, debris, and background biological impurities that affect detection assay performance for these types of samples. The E-PCR method is similar to the RV-PCR method [17], however, the incubation broth, incubation time, DNA extraction kit, and PCR method utilized were modified for optimal performance with soil matrices. The study utilized medium-to-low spikes of surrogate *B. anthracis* endospores (1350 to ~4.5 endospore spike range to cover mid- and lower-range detection limit issues) in 9 g samples using three soil types (loamy sand, sandy loam, and clay rich soils) to enable the sensitive detection of low numbers of viable endospores. This study also included a comparison of the E-PCR protocol under development to a colony forming unit spreadable (CFU-S) assay that was previously developed and published jointly between the U.S. Geological Survey (USGS) and the U.S. Environmental Protection Agency (USEPA) [15,19] to contrast the differences in limits of detection among the two assays. Finally, this study provided a preliminary examination into how clay soils can potentially influence *B. anthracis* activation in soil using a series of 9 gram pure and blended sterilized loamy sand and clay samples. This information provides an important consideration for future analysis of clay soil types.

## 2. Methods

### 2.1. Bacillus Endospores and Soil

A 3 mL stock suspension of ATCC 9372 *Bacillus atrophaeus* subsp. *globigii (B.g.)* endospores was provided by the USEPA for use in these spike-based experiments. Spread-plates were inoculated with 100–200 μL of a dilution series of this stock (in deionized and ultra-filtered H_2_O, onto tryptic soy agar plates (TSA) in duplicate and incubated at 36 °C for ~24 h) and were utilized to identify dilution levels that would allow endospore spikes of ~4.5, 45, 225, 675 and 1350 per sample.

Three soil sample types were obtained from Agvise Laboratories Inc. (Benson, Minnesota) and included a sandy loam (60% sand, 36% silt, 4% clay, 12.4% organic matter, pH 6.5, loam Lot 11082014-3 and referred to as “loam” for the remainder of the paper), a loamy sand (85% sand, 6% silt, 9% clay, 2.2% organic matter, pH 5.9, sandy Lot 12072014-3 and referred to as “sand” for the remainder of the paper) and a clay (27% sand, 24% silt, 49% clay, 2.2% organic matter, pH 8.1, clay Lot 05292015-3). The loam and sand soils were obtained from an area of southeastern South Dakota endemic to anthrax. The clay soil was obtained from a region in the western Dakotas where outbreaks of anthrax are not known to occur. Soil samples were autoclaved twice at ~121 °C for 55 min and allowed to cool to room temperature for several hours before use. A zero-endospore spike in each experimental group was utilized to determine the sterility of soil.

### 2.2. CFU-S Assay

This assay followed a previously published protocol using 9 grams of double-autoclaved soil [15,19]. In short, five 9-gram samples were spiked with ~4.5, 45, 225, 675 and 1350 endospores, respectively. A sixth 9-gram sample was utilized as a no-spike control. A duplicate set of samples was simultaneously prepared for use with the E-PCR assay, detailed below. The culture sample set was then placed on a vertical rotator and rotated overnight at room temperature. A series of soil suspension, centrifugation, and pellet suspensions were then utilized following the previously published protocol (which is described step by step in USEPA and USGS, 2017) to produce a 25 mL buffer suspension of the original 9 grams of soil. Two-hundred μL of the suspension was spread on each of 10 TSA spread-plates which were then incubated for ~24 h at 36 °C for CFU enumeration. Each spike experiment was conducted three times for each of the soil types.

### 2.3. E-PCR Assay

For the spiked sample set prepared for the enrichment-PCR assay, 25 mL of cold tryptic soy broth was added to each soil sample tube (5 tubes containing ~9 g soil spiked at either ~4.5, 45, 225, 675 or 1350 endospores and one 9 g unspiked soil sample used as a no-spike control) and vortexed briefly for ~5 s. The vortexed tubes were allowed to settle for 10 min and then a 1.25 mL subsample from each tube was transferred to a microfuge tube and stored at −20 °C (subsamples labeled as time zero sample set). The samples remaining in the original tubes were then incubated at 36 °C in a Boekel® (Boekel Scientific, Feasterville, PA) model 136400 hybridization incubator (rocker set at ~3/4 full speed) for ~24 h. At ~24 h, samples were briefly vortexed and left to settle for ~10 min prior to taking a 1.25 mL subsample which was stored at −20 °C (subsamples labeled as the 24 h incubation sample set).

To determine the minimal incubation period for enrichment, the first loam enrichment experiment included a 6 h incubation sample in addition to the time zero and the 24 h incubation time-points. To determine if additional incubation time would increase sensitivity, the subsequent loam experiment and first sand experiment included a 48 h time-point in addition to the time zero and the 24 h incubation time-points. The remaining experiments only included time zero and 24 h incubation time-points (once it was determined that this incubation period was optimal vs. the 6 and 48 h time-points).

DNA was extracted from each sample using 200 μL of each −20 °C subsample. The Qiagen® (Germantown, MD) DNeasy® Powersoil Kit was used for extractions with a modification of their protocol of eluting the DNA with 100 μL of Qiagen’s AE buffer (improves the stability of DNA in cryogenic storage) instead of the kit elution buffer. In order to confirm the DNA concentrations extracted from the samples, the Qubit Fluorometer was used to measure 10 μL of DNA extract following the Qubit assay protocol. The Qubit Fluorometer uses fluorescent dyes which fluoresce only when bound to strands of nucleic acid and readings were taken and reported as μg/mL. For PCR, 2 μL of a 1/10 dilution of the extracted DNA was utilized as template. All PCR reactions were carried out in duplicate and included an internal positive control ([IPC] TaqMan Exogenous Internal Positive Control kit, Applied Biosytems, Forster City, California). Reactions were judged positive if amplification was noted in both duplicates. The primer and FAM labeled probe sequences for the detection of *B.g.* were previously described [20]. The master mix recipe per 20 μL reaction was 10 μL of 2X TaqMan® Fast Universal Master Mix (Applied Biosystems, Foster City, California), 1 μL of a 10 μM mix of primers and probe, 2 μL of 10X Exogenous Internal Positive Control (Applied Biosystems, Foster City, California), 1 μL of 50X Exo Internal Positive Control DNA (Applied Biosystems, Foster City, California), 4 μL of PCR certified H_2_O, RNase and DNase free (Teknova, Hollister, California) and 2 μL of template. The PCR amplification profile was 60 °C for 30 s, 50 °C for 2 min, 95 °C for 5 min and then 45 cycles of 95 °C for 15 s and 60 °C for 1 min. Presence/absence PCR reactions were run in an Applied Biosystems® StepOne Plus qPCR thermocycler. For a sample to be called positive, cycles needed to cross an Rn (magnitude of the probe signal) of 0.1 (lower level threshold for sample to be called positive). Primers and the probe were validated with DNA extracted from a pure culture of *B. atrophaeus* subsp. *globigii*.

### 2.4. Sand Versus Clay and Soil Blend Experiments Utilizing Culture

To determine the potential influence of clays on endospore activation that were noted during the CFU-S experiments conducted during this study, a series of 9 gram pure and blended samples were prepared using sterilized sand and the clay samples (same sand and clay samples as utilized above). Ratios of the prepared samples were 100% sand/0% clay (~ concentrations = 85% sand, 6% silt, 9% clay and 2% organic matter), 80% sand/20% clay (7.2 g sand/1.8 g clay, ~ concentrations = 73% sand, 10% silt, 17% clay and 2% organic matter), 60% sand/40% clay (5.4 g sand/3.6 g clay, ~ concentrations = 62% sand, 13% silt, 24% clay and 2% organic matter), 40% sand/60% clay (3.6 g sand/5.4 g clay, ~ concentrations = 50% sand, 17% silt, 33% clay and 2% organic matter), 20% sand/80% clay (1.8 g sand/7.2 gclay, ~ concentrations 39% sand, 20% silt, 41% clay and 2% organic matter) and 0% sand/100% clay (~ concentrations = 27% sand, 24% silt, 49% clay and 2% organic matter). The samples were spiked with ~1350 endospores each and shaken overnight at room temperature. Samples were processed and enumerated using the previously described CFU-S assay. This experiment was conducted twice.

### 2.5. Statistical Analysis

Comparisons of the data for endospore recovery from 9 grams of soil (i.e., total CFU/10 plates) for the three soil types were performed using one way ANOVA with the Tukey HSD post-hoc analysis for determining which soils had significantly different recovery rates within and between the different soil types at each endospore spike concentrations. Additionally, this same approach to testing for significant differences within and between soil types was applied to the DNA concentration in PCR sample data as measured by the Qubit system. The DNA concentration data for the 24 h time point were normalized prior to statistical analysis by subtracting the DNA concentrations at the zero time point from the respective data at the 24 h time point. All comparisons were performed at an α = 0.05 using Minitab (version 17) (Minitab, LLC, State College, PA).

## 3. Results

### 3.1. CFU-S Assay

Table 1 lists the total CFU recovered from all inoculated TSA plates for each soil/spike set (three soils samples were used for each soil type and 10 replicate plates were used for each soil sample, totaling 30 plates per soil type). For samples which were not spiked, no CFU were detected on any of the plates. The highest spike concentration of 1350 endospores was the only spike level where at least 1 CFU was noted on every spread-plate for all three soil types. The 675 endospore spikes resulted in 2/30 loam, 1/30 sand and 1/30 clay plates where no CFU were detected. The 225-endospore spike sample set resulted in 10/30 loam, 10/30 sand and 2/30 clay plates where no CFU were detected. The 45-endospore spike sample set resulted in 27/30 loam, 28/30 sand and 16/30 clay plates where no CFU were detected. The ~4.5 endospore spike sample set resulted in 28/30 loam, 30/30 sand and 29/30 clay plates where no CFU were detected. Within soil type groups, the average CFU for 9 gram spiked samples (endospore spikes of 4.5, 45, 225, 675, and 1350) were: loam samples, 0.1, 0.1,1.0, 2.5, and 7.2 CFU respectively; sand samples, 0.0, 0.1,1.1, 3.7, and 6.2 respectively; and clay samples, 0.0, 0.5,2.4, 27.4 (7.5, if not counting the CFU anomaly observed in experiment 2), and 17.2, respectively.

### 3.2. Enrichment-PCR Assay

Table 2 lists the DNA concentrations for the time 0 and time 24 h enrichment samples. For the time 0 h measurements, nine of the 54 samples produced signal, including two of the samples that were not spiked. DNA concentration for this data ranged from 11.0 to 42.0 ng mL^−1^. None of these samples were PCR positive for *B.g.* For the time 24 h measurements, three of the nine samples that were not spiked produced signal that ranged from 12.0 to 67.0 ng mL^−1^. None of these nine-time zero samples were PCR positive. For the spiked sample set, 44 out of 45 samples produced DNA concentration data that ranged from 10.0 to 676.0 ng mL^−1^. The only spiked sample that did not produce signal detectable by the Qubit was the 4.5 endospore spike clay experiment number 2 (Table 2); however, this sample was PCR-positive (Tables 3 and 4). The lone sample in this spiked sample set that was PCR negative was the 4.5 endospore spike in the first loam experiment (Tables 3 and 4) and the Qubit concentration for this sample was 12.0 ng mL^−1^ (Table 2). Average Qubit readings for each soil type group were 93.8, 147.7, 97.6,49.8 and 63.0 ng mL^−1^ for the loam samples with endospore spikes of 1350, 675, 225, 45, and 4.5 per 9 g of soil respectively; 160.2, 227.0, 173.0, 169.7 and 211.0 ng mL^−1^ for the sand sets with endospore spikes of 1350, 675, 225,45, and 4.5 per 9 g of soil, respectively; and 380.7, 310.3, 267.7, 211.3 and 230.3 ng mL^−1^ for the clay sample sets with endospore spikes of 1350, 675, 225, 45, and 4.5 per 9 g of soil, respectively.

Three other data sets that were collected during the first three experiments included a 6 h time-point for the first loam experiment 1 and 48-h time-points for the loam experiment 2 and sand experiment 1 (data not shown). Time 6 h extracts produced Qubit concentration data that ranged from 0.0 to 16.0 ng mL^−1^ in four of the six samples but all were PCR negative. The 48 h incubation groups that resulted in increased DNA concentrations and fewer cycles (avg. ~23 versus the fastest 24 h at 27) needed to cross an Rn of 0.1. The loam experiment 2 cycle number to Rn 0.1 ranged from 22–24 and averaged 23.4. The Qubit DNA concentration for the data set was >285 ng mL^−1^ and averaged 895.8 ng mL^−1^ for all the spiked samples. The sand, experiment 1, cycle number to Rn 0.1 was 23 for all the spiked sample with a Qubit DNA concentration that was ≥438 ng mL^−1^ and averaged 554.0 ng mL^−1^. No PCR positives were noted for the samples that were not spiked.

Table 3 lists the PCR cycle to an Rn of 0.1 data for the post 24-h incubation samples. Samples that did not receive an endospore spike showed no PCR amplification for all three soil type sets. All of the spiked enriched samples were PCR-positive, with the exception of the 4.5 endospore spike in the sand experiment 1 reaction. Cycle threshold values to an Rn of 0.1 was 25.5 for all spiked samples for the loam experiment 1 (except the previously mentioned 4.5 endospore spike), 27 for all spiked samples for the loam experiment 2, and 23.5–27.0 for the spiked samples in loam experiment 3. Spiked sand experiment cycle numbers ranged from 26.0 to 35.0, 24.0to 26.0 and 24.0to 25.0 for experiments 1, 2 and 3 respectively. Spiked clay experiment cycle numbers ranged from 23.5 to 24.5, 25.0to 31.0 and 24.5to 25.0 for experiments 1, 2 and 3 respectively. Results for the IPC sample groups were: loam experiments 1, 2 and 3 were a control cycle number to an Rn of 0.1 of 28.5, 29 and 27.5 for the controls and 28.5, 30 and 29 for the samples respectively; sand experiments 1,2 and 3 were 29, 29 and 27 for the controls and 29,32–40 and 29 for the samples respectively; clay experiments 1,2, and 3 were 29, 28.5 and 27.5 for the controls and 29, 28.5 and 29 for the samples respectively (data not shown).

Table 4 compares the CFU-S assay data to the E-PCR data for the three soil types spiked at 0, 4.5, 45, 225, 675, and 1350 endospores for the post 24-h incubation samples in terms of a presence/absence format. In Table 4 the CFU-S assay data were labeled “+” (positive) if the CFU average for the 10-plate sample set was >1 CFU and “−” (negative) if the CFU was <1 CFU. PCR data were labeled as “+” for cycle thresholds times that were <= 36 and “−” for cycle threshold times >36. Based on these criteria, Table 4 shows that both methods reported an absence of endospores when no endospore spike was added to the sample. Both methods reported the presence of endospores for all three soil sample sets when a 675 or 1350 endospore spike/9 g soil was used. However, differences in the methods were noted for the 4.5, 45, and 225 endospore spikes. For the 4.5 endospore/9 g soil spike, all CFU-S assay samples were reported as negative, while 8/9 samples were reported as positive in the E-PCR method. For the 45 endospore/9 g soil spike, all CFU-S assay samples were reported as negative while all nine PCR samples were reported as positive. For the 225 endospore/9 g soil spike, seven/nine of the CFU-S assay samples were reported as positive while all nine of the PCR samples were reported as positive.

### 3.3. Sand Versus Clay and Soil Blend Experiments Utilizing Culture

Table 5 lists the data for the sand versus clay and soil blend experiments. In experiment 1, CFU ranges for 100% sand, 80% sand/20% clay, 60% sand/40%clay, 40% sand/60% clay, 20% sand/80% clay and 100% clay were 4–22, 5–16, 8–18, 9–18, 5–22 and 11–21, respectively. In experiment 2, CFU ranges were 8–14, 7–24, 6–17, 9–20, 10–17 and 7–17 for the 100% sand, 80% sand/20% clay, 60% sand/40%clay, 40% sand/60% clay, 20% sand/80% clay and 100% clay, respectively. Between the two experiments (column three in Table 5), the average CFU for the six sample types were 11.2, 12.4, 12.4, 13.8, 13.9 and 13.9 from 100% sand to 100% clay, respectively.

### 3.4. Statistical Analysis

The results of the statistical analysis are presented in Tables 6 and 7.

## 4. Discussion

### 4.1. CFU-S Assay

The only *B.g.* spike concentration for the CFU-S assay where CFU were detected on all plates was the 1350-endospore spike sample set. The 675-endospore spike was fairly consistent with regard to CFU detection, but within each soil type, there was at least one plate where CFU were not detected. For the both the 225- and 45-endospore spike experiments, CFU were detected on more plates from the clay soil than the other two soil types. However, for the 4.5-endospore spike plates, the majority of the plates were not detected for each soil type, which is most likely due to expected variability with low-end spikes. As was observed in a previous publication that reported a detection limit of 14 CFU g^−1^ using this culture-based assay [15], consistent average detection of at least 1 CFU g^−1^ occurred at the 25 endospore g^−1^ spike and was not detected at the 5 endospore g^−1^ spike in this data set.

It can be seen from Table 1 that in the 45- to 1350-endospore spike range, the concentration of CFU was greater in all cases in the clay soil. This could potentially indicate that some component in the clay soils causes endospore activation; however, further research would be needed to confirm this hypothesis. In previous unpublished laboratory experiments, this clay-induction was noted by the authors and on occasion, an abnormally high count was observed within a sample group, as can be seen in Table 1 for the 675-endospore spike in clay experiment 2. This is most likely due to variability of the concentration of the induction agent within the clay soil that was used for these experiments.

### 4.2. E-PCR Assay

As shown in Table 3, the E-PCR assay demonstrated the ability to consistently detect 45 endospore spikes. The E-PCR assay further demonstrated the ability to detect all but one of the ~4.5 endospore spikes. Early time series data illustrated that at 0 and 6 h of incubation, the spikes could not be detected and while the 48-h time-point required fewer cycles for effective amplification, the 24 h time-point resulted in sensitive amplification of the lowest spikes. Other research in the USGS author’s lab (unpublished) demonstrated the need for an assay that would effectively eliminate the inhibition of PCR and allow repeatable detection of the target organism. Amplifying the target organism through enrichment effectively negates the ability of inhibitors to shut down a reaction or produce sporadic signal in replicates. The IPC used in these experiments demonstrated that the effect of inhibition was negligible and had amplification curves that crossed an RN of 0.1 within the cycle range of 27.5–29 for the entire data set. The sample IPC was the same as the control for experiments loam 1, sand 1 and clays 1 and 2. The sample IPC was within 1.5 cycles for loams 2 and 3, and clay 3, within 2 cycles for sand 3, and exhibited a cycle difference of 3–11 for sand 2. Positive samples produced amplification curves that crossed an Rn of 0.1 with a range of 23.5–31 (Table 3). The majority occurred between cycles 23.5 and 27 with the exception of the sand experiment 1~4.5 endospore spike and the clay experiment 2,45 and ~4.5 endospore spikes which had cycles over 27.

### 4.3. Comparison of CFU-S and E-PCR Endospore Detection Methods

The most desired qualities of a microbial detection method when analyzing environmental samples are (1) the shortest time possible between sample collection and production of reliable results and (2) a low overall cost in terms of technicians and supplies. As outlined above, the CFU-S assay recovers the spiked endospores from the soil samples by processing the entire 9.0 g of soil into a single volume (25 mL) of extraction solution, then plating sub-samples (200 μL) onto individual TSA plates. Assuming that all endospores were homogeneously mixed throughout each soil sample and the extraction method was 100% efficient, the total number of endospores expected to be present on all of the 10 TSA plates inoculated with 200 μL each, bringing the total volume of the extraction solution plated to 2.0 mL, would be 8.0% of the original spike concentration (Table 1). The total number of endospores recovered (percent recovery) from all 10 plates for each soil type and spike concentration are listed in Table 1. Based off the data reported for total CFU for the 10 plates, the CFU-S method was insensitive or unreliable for recovering endospores at concentrations of approximately 4.5 endospores/9 grams of soil (Table 1). However, when the average of the 10 plates was considered in terms of presence/absence where the average CFU for the set was set at ≥1 to be considered positive (Table 4), then the CFU-S method was also unreliable at the 45 endospores/ 9 grams of soil spike.

The CFU-S method recovers 0–75.9% for the loam soil and 0–90.7% for the sand soils for the endospore spike concentrations of 45–1350 endospores/9 grams of soil. The percent recoveries in the clay soils were significantly greater (0 to >500%) than the same original spike concentrations. A higher recovery of endospores than what was originally inoculated was also observed by the authors of the current paper during a preliminary study (data not shown) which utilized *B. anthracis* Sterne strain endospores inoculated into clay-rich soils (the same 49% clay soil that was used in this study). This observation was not noted with the other two soil types (loam and sand) that were used. The findings in both this study which utilized *B.g.* endospores, and the preliminary laboratory study which utilized Sterne strain endospores, suggest that clay content in soil might have an effect on analysis results. Thus, the sand versus clay blended experiments were conducted to further investigate the findings.

The E-PCR method artificially increases the number of vegetative cells from the spike endospores by incubating the entire 9.0 g soil sample in 25 mL of TSB, then sub-sampling (200 μL) that incubation solution and processing that volume for recovery and purification of total DNA, which is suspended in a final 100 μL volume. Approximately 2.0 μL of this DNA solution is used for the PCR assay. The E-PCR method has many similarities to the RV-PCR method [17], however, the incubation broth, incubation time, DNA extraction kit utilized, and PCR method utilized for the E-PCR method were modified from what was used for RV-PCR (brain heart infusion broth, a 9-h incubation time, the Magnesil® Blood Genomic Max Yield System Kit (Promega, Madison, WI) and real-time PCR) for optimal performance with soil matrices. While recoveries for the E-PCR method are not directly comparable to recoveries for the RV-PCR method which has not been tested directly for a soil matrices, the E-PCR method had a similar sensitivity (detect the presence of endospores from all soil types with spikes of 4.5–1350 endospores/9.0 grams-Table 3) to that of RV-PCR (10 to 99 endospores for wipes, air filters, and water).

The E-PCR method cannot be directly compared to the CFU-S method. That is because there is an implicit condition for the culture-based method that no increase in the number of endospore or vegetative forms of those endospores occurs during the period between sample collection and plating. In the case of the PCR method, the enrichment step, which is designed to increase bacterial numbers, is the basis for the PCR method. However, when methods for the recovery and detection of bacteria from environmental samples are being compared to decide which is most appropriate for a specific objective, comparing those methods based on the sensitivity of the assay, the time interval between sample collection and the production of reliable data and the overall costs of the analysis are justifiable metrics.

Sensitivity of the method might be an important factor when deciding which method to use for applications of the method following decontamination of a site where the method is needed to reliably detect low numbers or no endospores remaining. When evaluating the sensitivity of the two methods, the averaged data for the CFU-S method must be assessed in a presence/absence format, where any value ≥ 1 is considered a positive (+) (Table 4). Using that criterion, the CFU-S method reliably detects the presence of endospores down to a concentration of 225 endospores/9 g of soil for all soil types. The E-PCR method detects the presence of endospores in all soil types at the lowest spike level of ~4.5 endospores/9 g soil (Table 4). In addition, none of the E-PCR samples were negative when the CFU-S samples were positive. Although enrichment of samples will allow germination of the endospores in order to increase bacterial numbers present in the sample if the target cells are present in the sample and viable, the method is not quantitative in nature and therefore, might not be appropriate if the exact starting concentrations in the sampled matrix are needed.

Comparison of the time required to process the samples and the cost of sample analysis might be an important factor in the decision of which analytical methods to utilize when large numbers of soil samples need to be analyzed. The CFU-S method described in this study requires the use of an extraction solution and procedure, the inoculation of multiple plates to reach a total plated volume to provide reliability for detection of low concentrations of endospores, and a time commitment of approximately 30 h (sample preparation, ~24 h incubation, enumeration and logging data). The estimated costs for this method, assuming 10 plates will be inoculated, is ~$10.00 per sample. The E-PCR method requires the suspension of the soil sample in 25 mL of TSB, the same incubation period as the CFU-S method, then the processing of a sub-sample of the enrichment solution to determine the presence or absence of endospores based on the presence or absence of their DNA. This method required slightly less time for completion than the CFU-S method (a time commitment of approximately 27 h for sample preparation, incubation, DNA extraction, PCR setup, DNA amplification and logging data), at an estimated cost of $15.00 per sample.

### 4.4. Sand Versus Clay and Soil Blend Experiments Utilizing Culture

To further investigate the effects of this clay soil sample on increased CFU recovery that was seen with the CFU-S clay results, several experiments using unblended and blended clay and sand were conducted (Table 5). The clay sample used in these experiments contains ~49% clay, as previously stated. As Table 5 illustrates, the CFU count with unmixed sand sample is an overall average of 11.2 CFU per agar plate and the CFU count with unmixed clay sample is an overall average of 13.9 CFU per agar plate. While the difference in the results between the sand and clay samples was not statistically significant, the CFU-S data in Table 1 show relative percent recovery rates that are greater than 100% for the 45 to 1350-endospore spike clay soils that were not observed with the loam or sand samples. The authors hypothesized that that the clay could potentially be causing endospore activation; however, additional experiments are needed to confirm this hypothesis and the study results.

The issue of the potential for clay-induced endospore activation is particularly interesting and in need of further research to explain why this endospore former is activating in clay-enriched soils but not sand or loam soils. In the environment, germination can occur in response to binding of nutrient germinants (purine nucleosides, sugars, amino acids) to germinant receptors (GR) located in the inner endospore membrane [21–23]. L-alanine and inosine are an example of co-germinants that can induce endospore germination in *B. anthracis* endospores [24–27]. Non-nutrient germinants, such as high concentrations of external CaDPA (a 1:1 chelate of Ca^2+^ and dipicolinic acid), can also trigger germination [21–23,28]. Compared to silt and sand particles, clay particles are smaller and therefore, have a larger surface area available for retention of moisture and nutrients. In addition, clay soils tend to carry a net negative charge which allows them to attract and adsorb positively charged ions such as Ca^2+^ [29]. The exosporium of *B. anthracis* endospores also carries a net negative charge when pH > 4.5 (becoming more negative with increasing pH and more alkaline soils), allowing the endospores to attract and adsorb calcium and other cations and potentially improve chances for germination to occur [5,30–33. Endospore germination and replication has typically not been found to occur in acidic soils [33]. The pH of the clay, loam and sand soils used in this study were 8.1, 6.5, and 5.9, respectively. Therefore, when the endospores were spiked into the clay-rich soil used in this study, the clay soil was potentially able to better retain nutrients needed for endospore germination and replication compared to the sandy or loamy soils. In addition, the higher pH of the clay could have contributed to the ability of the endospores to attract and absorb the nutrients needed for germination and replication to occur. However, this study did not specifically analyze for these nutrients in the soil samples utilized to verify this hypothesis. Interpretation of data resulting from analysis of clay soils might need to consider this potential for activation and how it might affect final estimates of recovery. Additional research is needed to understand what is causing the activation within the clay soils.

### 4.5. Statistical Analysis

In general, the recovery of the endospores (Table 1) at the lower spike concentrations (i.e., 0–225 endospores/9 grams of soil) was non-proficient, regardless of which soil type was processed as there were no significant differences in the respective recovery rates (Table 6). The higher endospore spike concentrations (i.e., 657 and 1350 endospores/9 grams of soil) were recovered at significantly greater rates when compared to lower spike concentrations, regardless of which soil was being processed. When comparing the endospore recovery rates for higher spike concentrations (i.e., 225, 675 and 1350 endospores/9 grams of soil) between the different soil types, there were no significant differences between the loam and sand soils. However, the clay soils had significantly greater endospore recovery rates than the loam and sand soils at these same spike concentrations (Table 7).

With regard to the DNA concentrations in the respective PCR reaction samples (Table 2), there were no significant differences between the concentrations within and between the soil types at the zero or the normalized 24-h time points, respectively.

## 5. Conclusions

The reproducibility and sensitivity of the CFU-S assay demonstrates a useful and robust protocol, but when working with unsterile samples, the ability to accurately enumerate *B. anthracis* specific CFU would be challenging even using selective growth media. This assay was originally developed to concentrate low numbers of endospores from soil matrices followed by identification using a molecular assay (PCR or PCR-variant, tagged antibody, etc.). The authors of this study originally looked at extractions of target DNA from the concentrated endospores followed by PCR (data not shown). These experiments illustrated that whatever commercially available kit we used for DNA purification, there was significant reaction inhibition. The only reliable and repeatable amplification that could be obtained was using concentrations of endospore spikes that were at least two logs greater than the highest spike concentration used in this study. The method that most easily addressed this obstacle was the adaptation of sample enrichment. This approach allowed endospore activation and vegetative growth to the point that at ~24 hrs incubation one can achieve sensitive and repeatable detection of very low numbers of endospores.

Given the tradeoff of endospore concentration for rapid kit extraction procedures and fast-cycling PCR reagents and thermocyclers, sample processing time is reduced by 3 h using the E-PCR assay compared to the CFU-S assay. The E-PCR assay is a good approach if the goal is the detection of low numbers of viable endospores. This would particularly be useful for remediation efforts and in determining the extent of dispersion during an incident resulting in soil contamination. A prime benefit of using an E-PCR approach is that it addresses endospore viability by increasing target cells present in the sample through enrichment if they are present and viable. Ultimately, if modified to an MPN-PCR format, then the assay would address viability and would provide a quantitative approach that is statistical in nature.

The potential activation and subsequent sporulation of endospores in clay soils might be an important consideration when processing and analyzing clay-based soil samples. Overall this clay related activation observation warrants further research. In addition, there is still a need for the development of an assay that is sensitive enough to reliably detect low numbers of endospores (as observed with this E-PCR assay) and is quantitative in nature (an MPN format).

## Figures and Tables

**Table 1.** Colony forming units spreadplate (CFU-S) assay data. Total colony forming units (CFU) recovered (% recovery) from all inoculated plates (n = 10).

Soils	Total CFU/10 Plates (Relative Percent Recovery)					

	(Number of Spiked Endospores/9 Grams Soil)					

	0	4.5	45	225	675	1350
**Loam 1**	0	0	2 (55.6)	10 (54.9)	28 (51.9)	72 (66.7)
**Loam 2**	0	2 (556)	1 (27.8)	9 (49.5)	14 (25.9)	61 (56.5)
**Loam 3**	0	1 (278)	0	12(66.7)	32 (59.3)	82 (75.9)
**Sand 1**	0	0	0	16 (89.9)	20 (37.1)	42 (38.9)
**Sand 2**	0	0	2 (55.6)	13 (71.4)	47 (87.0)	80 (74.1)
**Sand 3**	0	0	0	3 (16.7)	51 (90.7)	66 (59.3)
**Clay 1**	0	0	6 (166)	25 (138)	91 (169)	178 (165)
**Clay 2**	0	0	5 (139)	22 (121)	673 (>500)	176 (163)
**Clay 3**	0	1 (278)	5 (139)	24 (132)	59 (109)	159 (147)

Expected recovery totals using 10 spread plates of 200 ul for each spike level for all soil types—0 = 0, 4.5 = 0.36 total CFU, 45 = 3.6 total CFU, 225 = 18.2 total CFU, 675 = 54.0 total CFU, 1350 = 108.0.

**Table 2.** Qubit DNA concentration data in ng/ml for time = 0 and time = 24 h incubation for the liquid culture enrichment.

Time Point in Hours	Endospore Spike Number/9 g Soil	Loam Replicate (Rep.) 1	Loam Rep. 2	Loam Rep. 3	Sand Rep. 1	Sand Rep. 2	Sand Rep. 3	Clay Rep. 1	Clay Rep. 2	Clay Rep. 3
0	0	0.00*	0.00	0.00	18.00	0.00	0.00	21.00	0.00	0.00
0	4.5	0.00	0.00	0.00	0.00	0.00	0.00	33.00	0.00	0.00
0	45	30.40	0.00	0.00	0.00	0.00	0.00	42.00	0.00	0.00
0	225	0.00	0.00	0.00	36.00	0.00	0.00	0.00	0.00	0.00
0	675	0.00	0.00	0.00	11.00	0.00	0.00	0.00	0.00	0.00
0	1,350	0.00	0.00	0.00	27.00	25.00	0.00	0.00	0.00	0.00
24	0	12.00	0.00	47.00	0.00	0.00	67.00	0.00	0.00	0.00
24	4.5	12.00	64.00	113.00	55.00	283.00	295.00	534.00	0.00	157.00
24	45	56.50	55.00	38.00	10.00	197.00	302.00	421.00	13.00	200.00
24	225	24.90	146.00	122.00	19.00	156.00	344.00	530.00	33.00	240.00
24	675	17.00	56.00	370.00	15.00	318.00	348.00	676.00	39.00	216.00
24	1,350	37.30	119.00	125.00	19.00	30.50	431.00	615.00	134.00	393.00

* 0.00 = < 10.0 ng/mL. Polymerase chain reaction samples in ng/mL.

**Table 3.** Liquid culture enrichment-polymerase chain reaction (E-PCR) cycle threshold data to a Rn of 0.1.

Soils	Number of Spiked Endospores/9 Grams Soil					

	0	4.5	45	225	675	1350
**Loam 1**	No Amplification	No Amplification	25.5	25.5	25.5	25.5
**Loam 2**	No Amplification	27	27	27	27	27
**Loam 3**	No Amplification	25	27	25	23.5	25
**Sand 1**	No Amplification	35	27	27	26	27
**Sand 2**	No Amplification	24	25	26	25	25
**Sand 3**	No Amplification	25	24	24	24	24
**Clay 1**	No Amplification	24	24.5	24	23.5	24
**Clay 2**	No Amplification	31	29	27.5	27	25
**Clay 3**	No Amplification	25	24.5	24.5	24.5	24.5

**Table 4.** Colony-forming unit spreadplate (CFU-S) data versus liquid culture enrichment-polymerase chain reaction (E-PCR) data.

Soils	CFU vs. PCR					

	(Number of Spiked Endospores/9 Grams Soil)					

	0 (CFU/PCR)	4.5 (CFU/PCR)	45 (CFU/PCR)	225 (CFU/PCR)	675 (CFU/PCR)	1350 (CFU/PCR)
**Loam 1**	−/−	−/−	−/+	+/+	+/+	+/+
**Loam 2**	−/−	−/+	−/+	−/+	+/+	+/+
**Loam 3**	−/−	−/+	−/+	+/+	+/+	+/+
**Sand 1**	−/−	−/+	−/+	+/+	+/+	+/+
**Sand 2**	−/−	−/+	−/+	+/+	+/+	+/+
**Sand 3**	−/−	−/+	−/+	−/+	+/+	+/+
**Clay 1**	−/−	−/+	−/+	+/+	+/+	+/+
**Clay 2**	−/−	−/+	−/+	+/+	+/+	+/+
**Clay 3**	−/−	−/+	−/+	+/+	+/+	+/+

+ equals sample positive - CFU average of ≥1 CFU for the 10-plate sample set or PCR positive. – equals sample negative - CFU average of <1 CFU for the 10-plate sample set or PCR negative.

**Table 5.** Sand versus clay and soil blend culture experiments. Average colony forming unit (CFU) per 10 tryptic soy agar plate set.

Soil Composition	Experiment 1	Experiment 2	Average CFU of Experiment 1 and 2
100% sand	11.4	11	11.2
80% sand/20% clay	11	13.8	12.4
60% sand/40% clay	12.6	12.1	12.4
40% sand/60% clay	13.5	14	13.8
20% sand/80% clay	14.7	13	13.9
100% clay	14.4	13.4	13.9

Each 9-gram sample was spiked with 1350 *Bacillus atrophaeus* subsp. **globigii** endospores.

**Table 6.** Statistical comparison of endospore recovery rates in Table 1.

Spiked Endospores/9 Grams Soil		Loam (*p*-Value)	Sand (*p*-Value)	Clay (*p*-Value)

[Spike] 1	[Spike] 2			
0 endospores	4.5 endospores	NS*	NS	NS
0 endospores	45 endospores	NS	NS	NS
0 endospores	225 endospores	NS	NS	NS
0 endospores	675 endospores	0.002	0.008	0.008
0 endospores	1350 endospores	<0.001	<0.001	<0.001
4.5 endospores	45 endospores	NS	NS	NS
4.5 endospores	225 endospores	NS	NS	NS
4.5 endospores	675 endospores	0.003	0.008	0.008
4.5 endospores	1350 endospores	<0.001	<0.001	<0.001
45 endospores	225 endospores	NS	NS	NS
45 endospores	675 endospores	0.003	0.009	0.009
45 endospores	1350 endospores	<0.001	<0.001	<0.001
225 endospores	675 endospores	NS	0.060	0.060
225 endospores	1350 endospores	<0.001	0.001	0.001
675 endospores	1350 endospores	<0.001	NS	NS

* NS = no significant difference.

**Table 7.** Statistical comparison of recovery method per soil type.

Spiked Endospores/9 Grams Soil	Soil Comparison	*p*-Value	Recovery Relationship
225 endospores	Loam vs. Sand	NS *	NA *
	Loam vs. Clay	0.017	Clay > Loam
	Sand vs. Clay	0.020	Clay > Sand

675 endospores	Loam vs. Sand	NS	NA
	Loam vs. Clay	NS	NA
	Sand vs. Clay	NS	NA

1350 endospores	Loam vs. Sand	NS	NA
	Loam vs. Clay	<0.001	Clay > Loam
	Sand vs. Clay	<0.001	Clay > Sand

* NS = no significant difference, NA = not applicable.
